# How to Make Students Happy During Periods of Online Learning: The Effect of Playfulness on University Students’ Study Outcomes

**DOI:** 10.3389/fpsyg.2021.753568

**Published:** 2021-10-06

**Authors:** Yuqian Li, Fengfei Hu, Xiao He

**Affiliations:** ^1^School of Teacher Education, Nanjing Xiaozhuang University, Nanjing, China; ^2^School of Physical Education, Nanjing Normal University, Nanjing, China; ^3^School of Management, Shanghai University, Shanghai, China

**Keywords:** playfulness, sense of control, life satisfaction, school burnout, university students

## Abstract

University students’ study outcomes, their psychological wellbeing in particular, have been considered closely by both education researchers and practitioners. It is worth exploring ways to improve the quality of life of students, especially during the pandemic period when millions of students around the world have taken online courses at home. This paper tests the influence of playfulness on the levels of life satisfaction and school burnout of college students. To examine our hypotheses, we distributed our survey to 353 Chinese university students (mean age 20.10 years) in their online learning semester in 2020 due to the outbreak of COVID-19. Correlation analysis and path analysis were applied to analyze the data. The results show that playfulness positively relates to life satisfaction and negatively correlates to school burnout. Moreover, a sense of control mediates these relationships. Both theoretical and practical implications are discussed.

## Introduction

Various studies have revealed a growing interest in the mental health of university students (Hunt and Eisenberg, 2010; Kraft, 2011; Gulliver et al., 2019). Attending university from late adolescence to adulthood is a big change for young people (Arnett, 2000; Wintre and Yaffe, 2000; Lin, 2010). In this period in their lives, university students may feel negative emotions, like depression, anxiety (Mahmoud et al., 2012), loneliness, stress, and learning burnout (Lin and Huang, 2012; Stoliker and Lafreniere, 2015), which may have a negative impact on their school lives, such as decreased learning abilities, poor academic achievement (Benner, 2011), and even school dropout (Eicher et al., 2014). According to the National Center for Education Statistics (U.S. Department of Education, National Center for Education Statistics, 2018), only 60% of all college students in the United States complete their undergraduate studies. Moreover, university students are likely to suffer from mental problems (Russell et al., 2019), such as sleeping difficulties (Becker et al., 2018), deliberate self-harm (Gratz and Roemer, 2008), and even suicidal thoughts and behaviors (Mortier et al., 2018). Thus, the issue of university students’ life quality has become significant (Benjamin and Hollings, 1995), and studies focusing on the quality of life have greatly increased in the past two decades (Rapley, 2003; Sirgy, 2012; Murgaš, 2016). This seems more important since the beginning of 2020 after the outbreak of COVID-19. During the pandemic period, millions of students around the world were unable to study on college campuses. Instead, they had to take online courses, which may lead to negative impacts on their psychological wellbeing. It has become meaningful to explore ways to improve their quality of life and alleviate negative consequences during this hard time.

Wrosch and Scheier (2003) found that personality can influence life satisfaction through the way people deal with life circumstances. As a personality trait (Shen et al., 2014), playfulness refers to “the predisposition to frame (or reframe) a situation in such a way as to provide oneself (and possibly others) with amusement, humor, and/or entertainment” (Barnett, 2007, p. 955). Early playfulness research mainly focused on children (Bundy, 1997). Although adults and adolescents also like to play (Sutton-Smith, 1966; Guitard et al., 2005), it is less socially acceptable among adults (Lieberman, 2014) because playfulness does not meet the increasingly rational and pragmatic image expected of adults by the public, and it can appear to be unattractive and useless (Piaget, 1962; Olsen, 1981). However, in the past three decades, a great number of researchers have paid attention to the functions and benefits of playfulness in adults through health or productivity indicators, like tension release and improved performance in the workplace (Martocchio and Webster, 1992). Some studies show that playfulness enables adults to keep an open mind, find solutions to problems more easily, and accept failure (Guitard et al., 2005). It is helpful to establish a persistently fun and pleasant internal environment, called the playfulness climate (Zhou et al., 2019). Moreover, researchers have found a positive relationship between adults’ playfulness and quality of life (Proyer et al., 2010). The playfulness of university students has been discussed far less frequently and in less depth than in children. Therefore, it is worth exploring whether playfulness could promote the quality of life of university students.

Previous studies of the quality of life have mainly focused on adults, whereas research on the psychological wellbeing of youngsters, including general life satisfaction and reactions to schooling, is scarce (Epstein and McPartland, 1976). For university students, the time spent in school occupies a large part of their lives. We take life satisfaction (Peterson et al., 2005) and school burnout (Salmela-Aro and Tynkkynen, 2012) as two indicators of the quality of life of university students. Specifically, life satisfaction is a summary view of the individuals’ overall lives (Hofmann et al., 2014). Life satisfaction has become an important issue during the schooling of university students (Diener and Larsen, 1993). Therefore, life satisfaction is used as the first indicator of the quality of university students’ daily lives. Furthermore, it is demonstrated that playfulness not only has a positive relation with psychological health (Staempfli, 2007), but also influences an individual’s emotions (Fredrickson, 1998, 2001). Students may experience feelings of strain and chronic fatigue in school (Raiziene et al., 2014). School burnout is regarded as the manifestation of maladjusted behavior at school (Fiorilli et al., 2017). Therefore, school burnout is used as another indicator to evaluate the quality of life of students in school. Furthermore, these two indicators could measure the university students’ quality of life from positive and negative aspects. In conclusion, life satisfaction and school burnout could help to assess university students’ quality of life.

We also noticed the term “sense of control” is used to refer to the extent to which an individual can control his or her own actions and govern external events through these actions (Chambon and Haggard, 2012). A sense of control can generate positive psychological and behavioral consequences (Seligman, 1975; Skinner, 1995), and social psychologists make efforts to explore how individuals manage themselves to achieve a subjective sense of control in unpredictable circumstances (Landau et al., 2015). Obtaining a sense of control is considered an essential element of wellbeing (Leotti et al., 2010). It is a critical social issue (Thompson and Spacapan, 1991), and we especially need to pay close attention to the sense of control in university students. Prior research has proven that the greater an individual’s sense of internal control, the lower their depression (Pearlin et al., 1981; Benassi et al., 1988). Therefore, sense of control may have an impact on university students’ quality of life.

In summary, the previous literature has the following gaps: (1) as an important group for the future, university students’ life quality deserves more attention; (2) playfulness seems like a bad quality and the positive role it could play on the university students’ life quality has been neglected; (3) although several proxies of quality of life have been suggested, there is a lack of measures applicable to the specific situation of university students’ life quality; and (4) a sense of control has not been explored as a possible link between university students’ personality and quality of life. Therefore, the present study explores improvements to university students’ life quality by analyzing the interactions among playfulness, life satisfaction, and school burnout, as well as examining the mediation effect of the sense of control.

### Playfulness and Life Satisfaction

Playful individuals can transform virtually any environment to make it entertaining and joyful (Barnett, 2007). Fredrickson (1998) argues that playfulness can facilitate experiencing joy and has the capability of building one’s intellectual, physical, and social skills. It is evidenced that playfulness positively relates to wellbeing across all age groups (Proyer, 2014b). Specifically, playfulness can facilitate positive emotions. Proyer and Ruch (2011) find positive relationships between adult playfulness and positive characteristics, such as creativity, humor, and the appreciation of beauty and excellence. In particular, some researchers discovered that adult playfulness is positively associated with life satisfaction and quality of life (Proyer et al., 2010; Seligman, 2011; Proyer, 2012, 2013).

### Playfulness and School Burnout

School burnout can lead to mental health issues, such as low self-esteem, depression, and a higher risk of suicide (Costello et al., 2006; Orth et al., 2008; Jiang et al., 2010). This is more obvious for adolescents. Schaufeli et al. (2002) note that school burnout negatively affects several dimensions of schoolwork and later has an impact on an individual’s health. Luckily, playing could be an effective strategy employed against boredom (Bowman, 1987; Barnett, 2011). It is believed that playfulness at work can facilitate socializing and release tension among colleagues (Yu et al., 2007). Ping (2006) proves that playfulness has a positive relationship with tension reduction and cheerful climate in the workplace. Playfulness also positively relates to job satisfaction (Glynn and Webster, 1992). In their study, Guitard et al. (2005) qualitatively conducted interviews with 15 adults and identified that playfulness is an important function for burnout prevention. However, the question is raised whether the same relevance between playfulness and burnout appears in different areas of life (Proyer, 2014a). Based on the above literature, this paper infers that specifically in educational situations, playfulness is negatively related to school burnout.

### Playfulness and Sense of Control

Bundy (1997) suggests that playfulness includes four factors: internal control, intrinsic motivation, freedom to suspend reality, and framing, which means internal control appears as a component of playfulness. However, Knox (1996) believes that internal control is a consequence of play. Even being somewhat different, the above two opinions both suggest that there is a certain connection between playfulness and internal control. Guitard et al. (2005) believe that a sense of control results from playfulness, which is consistent with Knox’s (1996) opinion. Proyer (2014a) proposes that playfulness has seven functions, including mastery orientation, wellbeing, and others. This shows playfulness can help to master one’s life orientation, which connotes a sense of control (Chambon and Haggard, 2012).

H1: Playfulness is positively related to a sense of control.

### Sense of Control and Life Satisfaction

A sense of control plays a critical role in physiological and psychological health and has been a focal research topic for several decades (e.g., White, 1959; Mittal and Griskevicius, 2014). Thompson and Spacapan (1991) conclude that the knowledge of control has a theoretical and practical contribution to understanding the sense of wellbeing in many different areas of life. Empirical research has demonstrated that the sense of control could be used to predict happiness and life satisfaction (Creed and Bartrum, 2008). Prior research shows that upper class people acquire positive health outcomes due to their high sense of control levels (Johnson and Krueger, 2005). Those with a low sense of control are shown to be psychologically aversive (Pennebaker and Stone, 2004) in ways that could further erode the quality of life.

H2: Sense of control is positively related to life satisfaction.

### Sense of Control and School Burnout

A sense of control not only influences students’ life satisfaction, but also their school burnout. Job burnout is a common occurrence in professional situations (Maslach et al., 2001), and there are also many studies describing school burnout among high school and college students (Schaufeli et al., 2002). For instance, Salmela-Aro et al. (2008) observe that burnout happens during the transition from comprehensive school to senior high school. Kiuru et al. (2008) consider that school-related burnout is possibly caused by personal expectations of school results or expectations held by teachers or parents. In other words, school burnout occurs when students’ performance-related outcomes fail to meet the expectations of teachers, parents, or themselves. Walburg (2014) concludes that exposure to high levels of chronic stress can engender burnout through an emotional state of exhaustion, cynicism, and depersonalization; this means that stress is a major reason of burnout that often appears when demands exceed the internal and external resources of individuals (Folkman, 2013) when a sense of control is lacking. Silvar (2001) attributes school burnout to a group of factors, such as lack of control, excessive school demands, and absence of interpersonal relationships. Spector (2002) considered that a lack of control generates anxiety and leads to negative health consequences.

H3: Sense of control is negatively related to school burnout.

### Playfulness, Sense of Control, Life Satisfaction, and School Burnout

According to control theory, two primary elements help describe human behavior: a cognitive and an affective element (Carver and Scheier, 1981). For the cognitive element, when people acquire a high sense of control by being playful, they may feel satisfied about life because they perceive that their performance levels conform to goal standards. For the affective element, when students lose their sense of control in school without enough playfulness, they may feel different about their desired and actual performances, leading to the potential generation of school burnout. Therefore, we believe a sense of control may play a mediating role in the relationship between playfulness and life satisfaction/school burnout.

H4: Sense of control mediates the relationships between both (a) playfulness and life satisfaction and (b) playfulness and school burnout.

## Materials and Methods

### Participants and Procedures

Data used in this study were collected among the students from a national university in East China during the first semester of 2020. At that time, all the students were required to take online courses at home because of the outbreak of COVID-19. The translation and back-translation methods were used to make a questionnaire (Brislin et al., 1973). All questionnaire items were rated with a 7-point Likert scale.

We distributed our questionnaires and collected data online. Six research assistants initiated snowball sampling through their personal contacts, who were asked to distribute the survey link to encourage more students to participate in this research. This sampling strategy is widely applied in data collection and has been proven to be reliable and effective (Meyerson and Tryon, 2003; Madrid and Patterson, 2016). Eventually, 353 samples were collected.

The demographic information of the sample is as follows: 125 participants are male (35.5%) and 228 are female (64.5%); age ranges from 17 to 26, and they are 20.10 years old on average (*SD* = 1.84); most participants are freshman (46.7%); and income of most participants was from 1,001 to 2,000 yuan (62.9%).

### Measures

#### Playfulness

We adopted 9 items from the International Personality Item Pool (IPIP) version of the playfulness subscale of VIA-IS to assess participants’ playfulness. An example item is “Try to tease my friends out of their gloomy moods.” The Cronbach’s alpha of the scale is 0.88.

#### Sense of Control

Lachman and Weaver’s (1998) 12-item scale was used to measure participants’ sense of control by assessing two aspects (personal mastery and perceived constraints). Sample items include “I can do just about anything that I really set my mind to” and “Other people determine most of what I can and cannot do.” The utility and psychometric properties of the instrument have been validated in several studies (e.g., Prenda and Lachman, 2001; Jang et al., 2008; Duffy, 2010) and had good reliability and validity in the Chinese context (e.g., Chou and Chi, 2001). The Cronbach’s alpha of the scale is 0.82.

#### Life Satisfaction

Five items were used to measure the participants’ life satisfaction from the Satisfaction with Life Scale (SWLS) (Diener et al., 1985). A sample item is “In most ways my life is close to my ideal.” The scale has achieved good reliability and validity in different cultural contexts, including Chinese (Liang and Zhu, 2015), German (Glaesmer et al., 2011), and Brazilian (Gouveia et al., 2009). The Cronbach’s alpha of the scale in this study is 0.90.

#### School Burnout

A five-item exhaustion subscale from the Maslach Burnout Inventory—Student Survey (MBI-SS) (Schaufeli et al., 2002) were applied to measure school burnout. An example item is “I feel emotionally drained by my studies.” The scale has been used among Chinese college students (Zhang et al., 2007). The Cronbach’s alpha of the scale is 0.89.

#### Control Variables

Mirowsky (1995) discovered that age exerts influence on the sense of control. In addition, age, gender (Fugl-Meyer et al., 2002), and income (Boyce et al., 2010) affect life satisfaction. Beer and Beer (1992) maintain that grades have an impact on school burnout. Therefore, to alleviate the impact of exogenous variables, several control variables are included in this research, including gender, age, grades, and monthly income.

### Analytical Strategy

To test the hypotheses, we first computed descriptive statistics of all measures and then conducted correlation analysis followed by a path analysis with R.

## Results

### Descriptive Statistics and Correlations

The descriptive statistics and correlations results among playfulness, sense of control, and life satisfaction are listed in Table 1. Specifically, playfulness positively relates to sense of control (*r* = 0.25, *p* < 0.01) and life satisfaction (*r* = 0.28, *p* < 0.01), but negatively relates to school burnout (*r* = –0.14, *p* < 0.01). The correlation between sense of control and life satisfaction is positive (*r* = 0.24, *p* < 0.01). However, the correlation between sense of control and school burnout is negative (*r* = –0.54, *p* < 0.01). Moreover, school burnout is negatively correlated with life satisfaction (*r* = –0.15, *p* < 0.01).

**TABLE 1:** Descriptive statistics and correlations of all study variables.

	Mean	*SD*	1	2	3	4	5	6	7	8
1. Playfulness	5.02	1.03	–							
2. Sense of control	4.41	0.82	0.25**	–						
3. Life satisfaction	4.16	1.30	0.28**	0.24**	–					
4. School burnout	3.83	1.47	–0.14**	–0.54**	–0.15**	–				
5. Gender	1.65	0.48	–0.08	–0.09	–0.04	–0.08	–			
6. Age	20.10	1.84	–0.06	0.09	0.00	0.00	0.13*	–		
7. Grade	2.21	1.47	–0.07	0.07	–0.00	0.02	0.20**	0.89**	–	
8. Income	2.14	0.93	0.11*	0.04	0.12*	0.06	–0.11*	–0.01	0.01	–

N = 353. Gender: 1 = male, 2 = female. Grade: 1 = freshman, 2 = sophomore, 3 = junior, 4 = senior, 5 = postgraduate or others. Income (per month): 1 = under 1,000 yuan, 2 = from 1,001 to 2,000 yuan, 3 = from 2,001 to 3,000 yuan, 4 = from 3,001 to 5,000 yuan, 5 = more than 5,000 yuan. *p < 0.05; **p <0.01.

### Path Analysis

Path analysis was conducted with the “lavaan” package in R to test the hypothesized relationships between playfulness, sense of control, life satisfaction, and school burnout. According to Browne and Cudeck (1993) and Kline (1998), the hypothesized model indicates a very good fit to the data. Specifically, the fit indices, chi-square (χ^2^) was 24.15, degree of freedom (df) was 6 (χ^2^/df < 5), Comparative Fit Index (CFI) was 0.90 (≥0.90), Incremental Fit Index (IFI) was 0.91 (>0.90), Goodness-of-fit Index (GFI) was 0.96 (>0.90), and Standardized Root Mean Square Residual (SRMR) was 0.05 (< 0.08).

**FIGURE 1:**
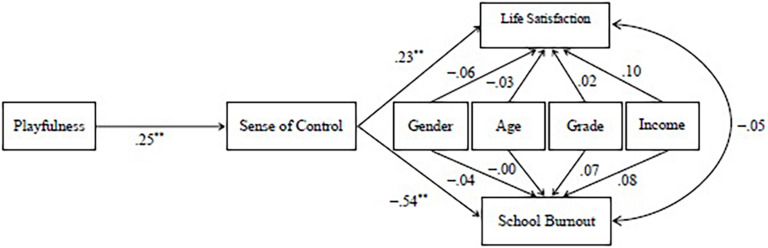
Path coefficients of the hypothesized model. *N* = 353. Standardized path coefficients are reported here. **p < 0.01.

Figure 1 illustrates the path coefficients of the hypothesized model. The results show that the path from playfulness to sense of control is significant (β = 0.25, *p* < 0.01), which supports Hypothesis 1. The paths from sense of control to both life satisfaction (β = 0.23, *p* < 0.01) and school burnout (β = –0.54, *p* < 0.01) are significant, so Hypotheses 2 and 3 are validated. In addition, according to Bond and Bunce (2003), the coefficients from playfulness to sense of control and from sense of control to life satisfaction are medium, and the coefficient from sense of control to school burnout is large.

Moreover, the results in Figure 1 also initially validate the mediating effect of a sense of control (H4) in the relationships between playfulness and life satisfaction/school burnout. The results described in Table 2 further illustrate the direct effect of playfulness on life satisfaction and school burnout and the indirect influences via sense of control. All the relationships are significant, which further supports Hypothesis 4.

**TABLE 2:** The direct and indirect effects of playfulness on life satisfaction and school burnout and 95% confidence intervals.

	Estimated effect (SE)	95% CI^a^
**Direct effects**		
Playfulness → Sense of control	0.25** (0.05)	[0.17, 0.33]
Sense of control → Life satisfaction	0.23** (0.05)	[0.08, 0.39]
Sense of control → School burnout	–0.54** (0.04)	[–0.70, –0.39]
Gender → Life satisfaction	–0.06 (0.05)	[–0.34, 0.22]
Gender → School burnout	–0.04 (0.05)	[–0.31, 0.24]
Age → Life satisfaction	–0.03 (0.11)	[–0.19, 0.13]
Age → School burnout	–0.00 (0.10)	[–0.16, 0.15]
Grade → Life satisfaction	0.02 (0.11)	[–0.18, 0.21]
Grade → School burnout	0.07 (0.10)	[–0.22, 0.26]
Income → Life satisfaction	0.10 (0.05)	[–0.04, 0.24]
Income → School burnout	0.08 (0.05)	[–0.06,0.21]
**Indirect effects**		
Playfulness → Sense of control → Life satisfaction	0.06** (0.02)	[0.01,0.10]
Playfulness → Sense of control → School burnout	–0.13** (0.03)	[–0.22, –0.05]

*N = 353.*

*^*a*^CI, confidence interval (1,000 bootstrap sample).*

*p < 0.05; **p < 0.01.

As for control variables, the results indicate that the paths from gender to life satisfaction (β = –0.06, n.s.) and school burnout (β = –0.04, n.s.), from age to life satisfaction (β = –0.03, n.s.) and school burnout (β = –0.00, n.s.), from grade to life satisfaction (β = 0.02, n.s.) and school burnout (β = 0.07, n.s.), and from income to life satisfaction (β = 0.10, n.s.) and school burnout (β = 0.08, n.s.) are non-significant.

## Discussion

This study uncovers the role of playfulness in enhancing university students’ quality of life during periods of online learning. In early 2020, a sample of 353 Chinese university students was selected for the study to examine the proposed hypotheses. The correlation analysis results show that students high in playfulness are likely to endorse a high sense of control. This study also reveals that a sense of control positively relates to life satisfaction and negatively relates to school burnout. In addition, the results of path analysis illustrate that a sense of control partially mediates the relationships between playfulness and life satisfaction and between playfulness and school burnout. Thus, our findings indicate that college students high in playfulness tend to possess a high sense of control, which can further enhance their life satisfaction and shield them from school burnout.

### Theoretical and Practical Implications

This research makes several theoretical and practical contributions. Theoretically, we identified the positive role that playfulness has on college students. Previously, playfulness was not regarded as a good quality (Lieberman, 2014), but rather a waste of time. Although a few prior studies recognized the contribution that playfulness can have on the wellbeing of individuals (Fredrickson, 2001), specific research aimed at university students is still scarce. This paper discovered that playfulness enhances the quality of life of university students, both physically and mentally. Second, this study proposes that life satisfaction and school burnout impact the quality of life of university students. These two indicators can be used in future research for measuring students’ quality of life. Third, this paper interprets the mechanism underlying the relationships between students’ playfulness and their life satisfaction/school burnout. Moreover, this study fills gaps by discovering the mediation effect of a sense of control. Hence, it provides a more detailed and specific framework to demonstrate how playfulness influences university students’ quality of life.

This study also has practical implications given that university students are an important cohort in society. We propose that society pay more attention to the spiritual qualities of university students’ lives, especially during the pandemic when students are taking online courses. This paper suggests that playfulness can enhance students’ sense of control to both enrich their life satisfaction and prevent school burnout; this indicates that developing playfulness could be meaningful in promoting the quality of life of university students. Therefore, the results from this study provide some guidance and advice for education practitioners. First, it is beneficial for schools to cultivate students who are psychologically happy and healthy. By promoting initiatives to help students train their sense of playfulness, universities could develop positive characteristics in the students. For example, schools can hold activities, such as meetings for sports or other games. Second, it benefits university students to know themselves to better manage their emotions and mentality. This paper suggests that university students should consciously strengthen their abilities to overcome difficulties to improve their quality of life. For instance, when experiencing setbacks, students need to learn to take advantage of playfulness (e.g., watching comedy movies) to adjust themselves. Moreover, students should cultivate their playfulness proactively in daily life, such as engaging in outdoor activities with friends.

### Limitations and Directions for Future Research

This study has several limitations. First, as a cross-sectional design study, the causal relationship between playfulness and quality of life cannot be validated. In the future, scholars should conduct longitudinal or experimental research to verify the findings. Second, all variable data were collected from the university students in a self-reported survey, which may give rise to improper conclusions. Hence, future researchers should collect data by other methods, such as interviews, behavioral observations, and experiments. Third, the source of the current sample was limited to students in one Chinese university, which leads to low generalizability. Future research should involve more diverse samples to generalize the current findings. Last, although the sample size of the current study is enough, it is suggested that a larger sample in future studies will further enhance the validity of empirical findings.

This research contributes to the literature in three ways. First, it makes great advances toward exploring the relationship among playfulness, sense of control, life satisfaction, and school burnout. Second, it provides a new thread for future research to regard the sense of control as a possible mediator when examining student-university relationships. Third, it digs into the relevance of individual personality and organizational environment to construct a foundation for future research of the interaction between individuals and different organizational environments. Obviously, university students are a dominant sector for the future of every country and finding effective ways to improve their quality of life is imperative.

## Data Availability Statement

The raw data supporting the conclusions of this article will be made available by the authors, without undue reservation.

## Ethics Statement

Ethical review and approval was not required for the study on human participants in accordance with the local legislation and institutional requirements. The ethics committee waived the requirement of written informed consent for participation.

## Author Contributions

YL and FH developed the theoretical framework and worked on literature review and manuscript writing. XH developed the theoretical framework and worked on data collection and analysis. All authors contributed to the article and approved the submitted version.

## Conflict of Interest

The authors declare that the research was conducted in the absence of any commercial or financial relationships that could be construed as a potential conflict of interest.

## Publisher’s Note

All claims expressed in this article are solely those of the authors and do not necessarily represent those of their affiliated organizations, or those of the publisher, the editors and the reviewers. Any product that may be evaluated in this article, or claim that may be made by its manufacturer, is not guaranteed or endorsed by the publisher.
